# Association between serum vitamin B12 and risk of all-cause mortality in elderly adults: a prospective cohort study

**DOI:** 10.1186/s12877-021-02443-z

**Published:** 2021-09-16

**Authors:** Kangjun Xu, Xiyu Liu, Jiaxin Liu, Yingying Zhang, Xiaohui Ding, Lin Li, Jiangwei Sun

**Affiliations:** 1 Department of Public Health, Shenyang Medical College, Liaoning, China; 2 School of Medical Sciences, Örebro University, Örebro, Sweden; 3 Department of Medical Epidemiology and Biostatistics, Karolinska Institutet, Stockholm, Sweden; 4 Institute of Environmental Medicine, Karolinska Institutet, Stockholm, Sweden

**Keywords:** serum vitamin B12, all-cause mortality, dose-response, cohort, CLHLS

## Abstract

**Background and purpose:**

Results from previous studies that linking vitamin B12 to risk of chronic diseases or mortality are inconsistent. We hereby explore the association between serum concentration of vitamin B12 and all-cause mortality risk in elderly adults.

**Methods:**

Participants aged over 65 years in the Chinese Longitudinal Healthy Longevity Survey were included in present prospective cohort study. Serum vitamin B12 was assessed at the 2011–2012 and 2014 wave, respectively. Participants were divided into three groups based on two cut-off points − 10th and 90th percentiles of vitamin B12 concentrations - in the whole population. Cox regression model was used to calculate the hazard ratio (HR) and 95 % confidence intervals (95 % CIs), and restricted cubic spline function was further modelled to investigate their dose-response associations.

**Results:**

Among 2,086 participants [mean ± SD: 87.74 ± 11.24 years, 908 (43.53 %) males], 943 (45.21 %) died during an average follow-up of 3.34 (SD: 1.63) years. Comparing with participants with middle concentration of serum vitamin B12, participants with high concentration had an increased risk of all-cause mortality [HR (95 %CIs): 1.30 (1.03–1.64)], whereas participants with low concentration had an insignificantly decreased risk of all-cause mortality (0.96, 0.76–1.20). The positive association between high concentration of serum vitamin B12 and all-cause mortality was also observed among the male and in a series of sensitivity analyses. In the dose-response analysis, a J-shape pattern was observed, but the non-linear association was only significant in males (*P*_non−linearity_ = 0.0351).

**Conclusions:**

High concentration of serum vitamin B12 was associated with an increased risk of all-cause mortality in a J-shaped pattern. The precise mechanisms underlying the association remain to be explored.

**Supplementary Information:**

The online version contains supplementary material available at 10.1186/s12877-021-02443-z.

## Introduction

As aging, the elderly population (aged more than 65 years) is expected to increase to more than 1.5 billion around the world by 2050 [1, 2]. The estimated number in China will be 400 million, 150 million of whom will be over 80 years [3]. China will encounter formidable medical challenges brought about by aging.

Vitamin B12 (cobalamin) is an essential hydro-soluble micronutrient to maintain health, which can be obtained from the diet (e.g., meat, eggs, and dairy products), supplements and intramuscular injections [4, 5]. It serves as a coenzyme for two metabolic enzymes: methionine synthase and methylmalonyl-CoA mutase. Inactivity of the methionine synthase enzyme could lead to the reduction in methylation reaction and accumulation of homocysteine concentrations in the tissues and serum, while loss of methylmalonyl-CoA mutase activity could induce the increase of serum concentrations of methylmalonic acid [6, 7]. Previous studies have shown that abnormal concentrations of vitamin B12 may be associated with various physiological dysfunctions or diseases. Its deficiency can lead to clinical symptoms such as macrocytic anemia and dementia [8–10], which commonly occur among elderly adults [11]. Multiple causes of low/deficient vitamin B12 status have been proposed, including malabsorption due to gastrointestinal dysfunctions (e.g., atrophic gastritis, coeliac disease, Crohn’s disease, or gastric/intestinal resection) [6, 12, 13], inadequate dietary intake (e.g., low dietary intake or vegetarianism), alcohol abuse, or medication use (e.g., metformin, proton-pump inhibitors or H2-receptor antagonists) [9, 14]. High concentration of vitamin B12 may be caused by liver or renal dysfunction [15, 16], which was associated with an increased risk of cardiovascular diseases (CVD) and cancers [17–19]. Based on these findings, we hypothesized that either low or high concentration of vitamin B12 is associated with an elevated risk of all-cause mortality in elderly adults.

Although the association between serum vitamin B12 concentration and all-cause mortality has been explored in previous studies [14, 20–23], the results are inconsistent: some studies reported a positive association between high vitamin B12 status and mortality in hospitalized older patients [14, 21] or the general population [22], while others not [23]. Moreover, there were some unaddressed issues. First, the enrolled population was mainly young adults, so the relationship between serum vitamin B12 concentration and all-cause mortality was still unclear among the oldest-old population (aged more than 80 years). Second, previous studies were mainly conducted in western countries, where dietary patterns differ from a Chinese diet, little is known about such association in detail among elderly adults in China. Third, serum vitamin B12 was usually transformed into a categorical variable in previous studies, therefore the dose-response relationship between them has not been well studied.

To this end, by using data from the Chinese Longitudinal Healthy Longevity Survey (CLHLS), we aimed to explore the relationship between serum vitamin B12 and risk of all-cause mortality among elderly adults.

## Methods

### Study design

More detailed information about CLHLS has been previously published [24]. Briefly, CLHLS is a nationally representative survey conducted in half of the counties and cities that were randomly selected in 22 provinces in China [25]. Established in 1998, the CLHLS conducted seven consequent surveys in 2000, 2002, 2005, 2008–2009, 2011–2012, 2014, and 2018–2019, respectively. Because vitamin B12 was only measured in 2011–2012 and 2014 wave, respectively, we therefore chose the 2,778 participants enrolled in those two waves as our studied population. We excluded participants who immediately lost to follow up after baseline survey (n = 464), who had missing information on birth date or end of follow up time (n = 93), who were younger than 65 years at baseline (n = 111), and those that had missing value in vitamin B12 (n = 24). Eventually, 2,086 participants were included (Sfigure 1).

### Biomarker measurement and outcome assessment

Fasting blood sample was collected from each participant by the trained medical personnel and centrifuged within 1 h after collection. The centrifuged serum was then stored at -20 °C and shipped to the central laboratory at the Capital Medical University in Beijing. We stratified the participants into three groups based on the following criteria: a). low concentration group, participants whose vitamin B12 concentration was less than 10th percentiles of its distribution (≤ 203 ng/mL); b). middle concentration group, participants whose vitamin B12 concentration ranged from 10th to 90th percentiles of its distribution (203–740 ng/mL); c). high concentration group, participants whose vitamin B12 concentration was more than 90th percentiles of its distribution (> 740 ng/mL).

Date of death was confirmed via the family member or the village doctor. Mortality from special causes (e.g., cancer, CVD) was not considered in present study due to two reasons: a). elderly adults mainly died at home, so cause of death was unclear; b). cause of death reported by family members may be not fully reliable [3]. All participants were followed from the enrollment until death, censoring, or 31 July 2019, whichever came first.

### Statistical analyses

Baseline characteristics were compared using an analysis of variance for continuous variables and ordinal Chi-square test for categorical variables. Post-hoc test was further performed to explore the difference between multiple groups. Cox regression models with baseline age as the underlying time scale were applied to explore the association between serum concentration of vitamin B12 and all-cause mortality risk. Four models with adjustment of different potential confounders were performed. We included age and sex (male/female) as covariates in model 1, and further adjusted for blood testing year (2011 or 2014), province of residence (categorical), residence (city, town, and rural area), ethnicity (Han/others), marriage status (married/others), occupation (farmer or manual, clerical, professional, and others), access to medical service (yes/no), smoking status (never, ever, and current smoker), drinking status (never, ever, and current drinker), exercise status (never, ever, and current exerciser) in model 2. In model 3, we further adjusted for vitamin supplementation use (yes/no), activities in daily living (ADL) score (categorical: 6, 5, 3–4, and 0–2), physical performance score (categorical: 5, 2.5–4.5, and 0-2.5), Mini-Mental State Examination (MMSE) score (an index for cognitive impairment, categorical: 24–30, 18–23, and 0–17), food diversity score (categorical: 6–8, 4–5, and 0–3), social activity score (categorical: 5–8, 3–4, and 0–2), and chronic disease score (categorical: 0, 1–2, and ≥ 3). The definitions of the above scores were described in our previous study [26]. In model 4, we additionally adjusted for clinical biomarkers including hemoglobin (continuous, associated with serum concentration of vitamin B12), total cholesterol (categorical, an index for hyperlipidemia), triglyceride (categorical, another index for hyperlipidemia), blood pressure (BP, categorical, an index for hypertension), glucose (categorical, an index for diabetes), and estimated glomerular filtration rate (categorical, eGFR, an index for renal function). The classification criterion of the above biomarkers was described in Stable1. The proportional hazard assumption of Cox regression model was evaluated via Schoenfeld residual plots and no violation was observed. Collinearity diagnostic was evaluated by the variance inflation factor and no collinearity among the covariates was found.

Subgroup analyses stratified by sex were performed to explore whether the association was homogeneous among males and females. To test the robustness of the results, we also conducted a series of sensitivity analyses among: a). subjects without vitamin supplementation use; b). subjects with high food diversity score; c). subjects without high total cholesterol; d). subjects without high triglyceride; e). subjects without high glucose; and f). subjects without renal dysfunction.

To assess the dose-response association between serum concentration of vitamin B12 and risk of all-cause mortality, vitamin B12 was modeled via a restricted cubic spline function with knots being set at 10th, 50th, and 90th percentiles of its distribution in model 4. The reference value for vitamin B12 was chosen as 450 ng/mL. We also explored whether the aforementioned knot selection was arbitrary by adding sensitivity analyses using different knots selection, including knots being set at “25th, 50th, 75th ”, “5th 25th 75th 95th ”, or “5th 25th 50th 75th 95th ” percentiles of its distribution. We observed that compared with the results from other knot sets, the result of selecting 10th, 50th, and 90th percentiles as the knot set was robust, as shown in Sfigure 2.

Data analyses were performed using SAS version 9.4 (SAS Institute Inc, Cary, NC) and R platform. A two-sided *P* less than 0.05 was considered significant.

## Results

The baseline characteristics of the enrolled 2,086 participants are presented in Table 1. The mean (standard deviation, SD) age was 87.74 (11.24) years and 908 (43.53 %) were male. Compared with participants with middle concentration of vitamin B12 (203–740 ng/mL), participants with high concentration of vitamin B12 (> 740 ng/mL) were likely to be younger, had higher level of social activity score and total cholesterol concentration, but lower level of ADL score, MMSE score, physical performance score, triglyceride concentration, systolic BP, and diastolic BP at baseline. No significant difference was found for sex, residence, ethnicity, marriage status, occupation, education concentration, smoking, drinking, vitamin supplementation use, chronic disease score, hemoglobin, glucose, and eGFR.

**Table 1:** Baseline characteristics by the levels of serum concentration of vitamin B12, a cohort study in China, 2012–2019

		Serum concentration of vitamin B12											
Variables		Low level (≤ 203 ng/mL)			Middle level (203–740 ng/mL)			High level (> 740 ng/mL)			*P* value		
No. of participants		210			1670			206					
No. of death		105			740			98					
Age at baseline, Mean ± SD, years		90.74 ± 10.54			87.57 ± 11.28			86.11 ± 11.14			< 0.0001 ^a b^		
Categories, n(%)													
< 80 years		39 (18.57)			455 (27.25)			60 (29.13)					
80–89 years		54 (25.71)			438 (26.23)			64 (31.07)					
90–99 years		57 (27.14)			409 (24.49)			51 (24.76)					
≥ 100 years		60 (28.57)			368 (22.04)			31 (15.05)					
Male, n(%)		87 (41.43)			732 (43.83)			89 (43.20)			0.6908		
Ethnicity (Han), n(%)		168 (98.82)			1308 (98.42)			149 (98.03)			0.8653		
Married, n(%)		143 (68.75)			1020 (62.50)			123 (62.76)			0.4669		
Residence, n(%)											0.0969		
City		7 (3.33)			56 (3.35)			12 (5.83)					
Town		16 (7.62)			245 (14.67)			27 (13.11)					
Rural area		187 (89.05)			1369 (81.98)			167 (81.07)					
Occupation, n(%)											0.7919		
Farmer or manual		174 (92.55)			1247 (88.25)			128 (87.07)					
Clerical		9 (4.79)			77 (5.45)			10 (6.80)					
Professional		2 (1.06)			43 (3.04)			5 (3.40)					
Others		3 (1.60)			46 (3.26)			4 (2.72)					
Education, n(%)											0.6738		
Illiterate		149 (73.40)			1073 (65.71)			121 (60.20)					
Primary school		45 (22.17)			429 (26.27)			61 (30.35)					
Middle school or above		9 (4.43)			131 (8.02)			19 (9.45)					
Smoking status, n(%)											0.8888		
Never		162 (78.26)			1255 (76.57)			146 (73.74)					
Ever smoker		16 (7.73)			133 (8.11)			23 (11.62)					
Current smoker		29 (14.01)			251 (15.31)			29 (14.65)					
Drinking status, n(%)											0.8806		
Never		167 (81.07)			1289 (78.89)			146 (75.26)					
Ever drinker		9 (4.37)			89 (5.45)			20 (10.31)					
Current drinker		30 (14.56)			256 (15.67)			28 (14.43)					
Exercise status, n(%)											0.7463		
Never		172 (83.50)			1322 (82.42)			163 (83.59)					
Ever exerciser		2 (0.97)			42 (2.62)			4 (2.05)					
Current exerciser		32 (15.53)			240 (14.96)			28 (14.36)					
Access to medical service, yes, n(%)		193 (93.24)			1577 (95.75)			188 (95.43)			0.4252		
Vitamin supplementation use, n(%)		31 (14.90)			200 (12.14)			25 (12.63)			0.5460		
ADL score, Mean ± SD		5.22 ± 1.66			5.42 ± 1.42			5.23 ± 1.69			0.0591		
MMSE score, Mean ± SD		18.22 ± 10.37			21.49 ± 9.46			21.29 ± 10.17			< 0.0001 ^a b^		
Physical performance score, median(IQR)		4.50 (3.00–5.00)			5.00 (3.50-5.00)			4.75 (3.50-5.00)			0.0293 ^a^		
Food diversity score, Mean ± SD		4.05 ± 1.86			4.35 ± 1.89			4.39 ± 1.82			0.0853		
Social activity score, Mean ± SD		1.91 ± 1.55			2.37 ± 1.68			2.52 ± 1.76			0.0003 ^a b^		
Chronic disease score, Mean ± SD		0.98 ± 1.86			0.87 ± 1.57			0.87 ± 1.23			0.8614		
Hemoglobin, median(IQR), g/L		121 (109–134)			123 (111–136)			123 (111–134)			0.1517		
Total cholesterol, median(IQR), mmol/L		3.89 (3.41–4.60)			4.49 (3.91–5.16)			4.69 (4.09–5.53)			< 0.0001 ^a b c^		
Triglyceride, median(IQR), mmol/L		0.82 (0.60–1.14)			0.91 (0.67–1.28)			0.86 (0.60–1.23)			0.0069 ^a^		
Systolic BP, Mean ± SD, mm Hg		140.01 ± 22.66			143.60 ± 25.01			139.47 ± 22.87			0.0167 ^c^		
Diastolic BP, Mean ± SD, mm Hg		79.39 ± 13.55			80.16 ± 13.57			77.66 ± 11.45			0.0360 ^c^		
Glucose, median(IQR), mmol/L		4.79 (4.15–6.16)			4.68 (4.04–5.48)			4.79 (3.96–5.48)			0.1471		
eGFR, median(IQR), mL/min/1.73 m^2^		67.05 (53.87–78.47)			67.89 (54.43–79.42)			68.17 (55.38–80.34)			0.5890		

Abbreviations: ADL: activities of daily living; BP: blood pressure; eGFR: estimated glomerular filtration rate; IQR, interquartile range; MMSE: Mini-Mental State Examination; SD standard deviation.

*P* value: analysis of variance for continuous variables; ordinal Chi-square test for categorical variables. ^a^ Statistical significance between low level group and middle level group; ^b^ Low level group and high level group; ^c^ Middle level group and high level group

**Fig. 1:**
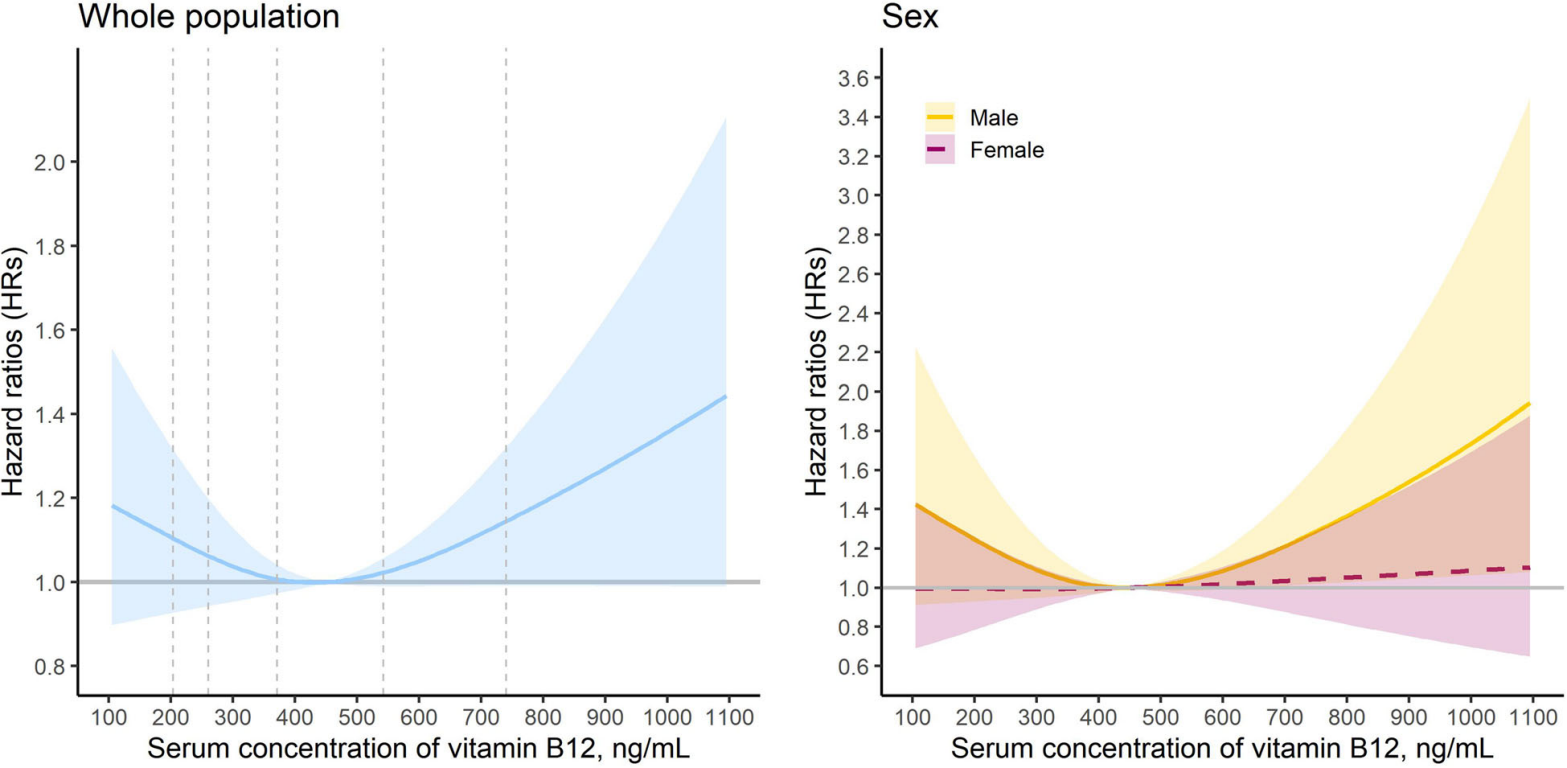
The adjusted dose-response associations between serum concentration of vitamin B12 and risk of all-cause mortality among the whole population, male and female, based on model 4. Serum concentration of vitamin B12 was modeled via a restricted cubic spline function with knots being set at 10th, 50th, and 90th percentiles of its distribution. The reference value was set at 450 ng/mL. The five vertical reference lines were used to denote the 10th (203 ng/mL), 25th (260 ng/mL), 50th (371 ng/mL), 75th (542 ng/mL), and 90th (740 ng/mL) percentiles of serum concentration of vitamin B12 among the whole population

A total of 943 (45.21 %) participants died during an average follow-up of 3.34 (SD: 1.63) years. We observed a robust association between vitamin B12 and all-cause mortality from model 1 to model 4. Compared with participants with middle concentration of serum vitamin B12, participants with high concentration had an increased risk of all-cause mortality (adjusted HR in model 4: 1.30, 95 % CI: 1.03–1.64), whereas participants with low concentration had an insignificantly decreased risk (0.96, 0.76–1.20) (Table 2). In the subgroup analysis stratified by sex, the positive association of high concentration of vitamin B12 existed in males (1.48, 1.03–2.13), but not in females (1.19, 0.86–1.64) (Table 2). In the dose-response analysis, a J-shape association between them was observed (Fig. 1). However, the non-linear association was only significant in males (*P*_non−linearity_=0.0351), not in the whole population and females (*P*_non−linearity_=0.0847 and 0.8653, respectively) (Fig. 1).

**Table 2:** Association between serum concentration of vitamin B12 and risk of all-cause mortality, a cohort study in China, 2012–2019

Population	Groups	Cases/Person-years	HR (95 % CIs)			
			Model 1	Model 2	Model 3	Model 4
Whole Population	Low level	105/671	0.99 (0.81–1.22)	1.10 (0.89–1.36)	1.03 (0.83–1.28)	0.96 (0.76–1.20)
	Middle Level	740/5633	1(Ref.)	1(Ref.)	1(Ref.)	1(Ref.)
	High level	98/670	1.33 (1.08–1.64)	1.25 (1.01–1.55)	1.27 (1.01–1.59)	1.30 (1.03–1.64)
Male	Low level	39/294	1.08 (0.77–1.52)	1.10 (0.77–1.56)	1.06 (0.74–1.52)	0.96 (0.66–1.41)
	Middle level	270/2573	1(Ref.)	1(Ref.)	1(Ref.)	1(Ref.)
	High level	45/292	1.59 (1.15–2.19)	1.41 (1.01–1.97)	1.42 (0.99–2.02)	1.48 (1.03–2.13)
Female	Low level	66/377	0.96 (0.74–1.24)	1.12 (0.86–1.47)	1.02 (0.77–1.34)	0.91 (0.68–1.22)
	Middle level	470/3060	1(Ref.)	1(Ref.)	1(Ref.)	1(Ref.)
	High level	53/378	1.16 (0.87–1.54)	1.15 (0.85–1.55)	1.17 (0.86–1.60)	1.19 (0.86–1.64)

Abbreviations: CIs: confident intervals; HR: hazard ratio

Model 1: adjusted for age and sex; Model 2: adjusted for model 1, enrollment year, province, residence, ethnicity, marriage status, occupation, access to medical service, smoking status, drinking status, and exercise status; Model 3: adjusted for model 2, vitamin supplementation use, ADL score, physical performance score, MMSE score, food diversity score, social activity score, and chronic disease score; Model 4: adjusted for model 3, hemoglobin, total cholesterol, triglyceride, blood pressure, glucose, and eGFR

The positive association of high concentration of vitamin B12 with all-cause mortality was also observed in the sensitivity analyses after excluding subjects with vitamin supplementation use (1.26, 0.99–1.62), low food diversity score (1.77, 1.05-3.00), high total cholesterol (1.28, 1.01–1.63), high triglyceride (1.32, 1.06–1.66), and abnormal eGFR (1.35, 1.00-1.81) (Table 3).

**Table 3:** Sensitivity analyses of the association between serum concentration of vitamin B12 and risk of all-cause mortality

Sensitivity analyses							Low Level			Middle Level			High level		
Subjects without vitamin supplementation use				Cases/Person-years			88/540			626/4776			80/560		
				HR (95 % CIs)			1.03 (0.81–1.30)			1(Ref.)			1.26 (0.99–1.62)		
Subjects with high food diversity score				Cases/Person-years			21/187			199/1713			24/182		
				HR (95 % CIs)			0.81 (0.49–1.33)			1(Ref.)			1.77 (1.05-3.00)		
Subjects without high total cholesterol				Cases/Person-years			99/652			691/5206			84/588		
				HR (95 % CIs)			0.94 (0.75–1.18)			1(Ref.)			1.28 (1.01–1.63)		
Subjects without high triglyceride				Cases/Person-years			100/652			699/5167			95/643		
				HR (95 % CIs)			0.97 (0.77–1.21)			1(Ref.)			1.32 (1.06–1.66)		
Subjects without high glucose				Cases/Person-years			78/590			665/5073			81/607		
				HR (95 % CIs)			0.86 (0.67–1.10)			1(Ref.)			1.22 (0.96–1.56)		
Subjects with normal eGFR				Cases/Person-years			55/448			397/3850			58/460		
				HR (95 % CIs)			0.92 (0.67–1.25)			1(Ref.)			1.35 (1.00-1.81)		

Abbreviations: CIs: confident intervals; HR: hazard ratio

The HR (CIs) was calculated based on model 4, which adjusted for age, sex, enrollment year, province, residence, ethnicity, marriage status, occupation, access to medical service, smoking status, drinking status, exercise status, vitamin supplementation use, ADL score, physical performance score, MMSE score, food diversity score, social activity score, chronic disease score, hemoglobin, total cholesterol, triglyceride, blood pressure, glucose, and eGFR

## Discussion

In this population-based cohort study, we observed that serum vitamin B12 was associated with the risk of all-cause mortality in a J-shaped pattern after adjustment for covariates, and the positive association of high concentration of vitamin B12 with all-cause mortality remains among males and in a series of sensitivity analyses.

Our findings that elevated serum vitamin B12 concentration was associated with a higher risk of all-cause mortality were consistent with the results from previous studies [15, 22]. Using data from the Newcastle 85 + study in the North East England, Mendonca et al. [15] found that women with higher plasma vitamin B12 concentrations had an increased risk of all-cause mortality (1.10, 1.04–1.16) and CVD mortality (1.10, 1.02–1.18) after adjustment for potential confounders, but similar associations were not observed among male. Flores-Guerrero and colleagues, identifying 5,571 participants (53.5 ± 12.0 years) from the PREVEND study [22], observed that per 1-SD increase of plasma vitamin B12 was related with a higher risk of all-cause mortality (1.25, 1.06–1.47). On the contrary, one study including 1,117 participants (75.1 ± 6.4 years) from a sub-study of the Longitudinal Aging Study Amsterdam reported no associations between vitamin B12 and risk of all-cause mortality in both men and women [23]. The underlying reasons for such inconsistency might include methodological limitations, different study populations (e.g., ethnicity or dietary habits), and methods for categorizing exposure groups. Compared with previous studies, we additionally applied the restricted cubic spline function in the Cox regression model and observed a J-shaped pattern between serum vitamin B12 and all-cause mortality risk. Although a similar dose-response pattern was reported previously, the non-linearity test of the association was not explored [15].

The precise underlying mechanisms of the association between elevated serum vitamin B12 and mortality have not been fully established, some explanations however have been proposed. Firstly, increased liver cell renewal and damage (caused by liver diseases) may disrupt its reabsorption by the liver (the biggest reservoir of vitamin B12 in the body), which alternatively increases its leakage from the liver [15, 16]. Second, upregulation of haptocorrin and transcobalamin synthesis or shifted binding affinity of transport proteins for vitamin B12, as consequences of chronic renal failure, hematological disorders [22, 27] or genetic polymorphism for encoding transcobalamin [28], can cause a high serum vitamin B12 status as well. Third, infectious diseases and cancers can also lead to the increase of serum vitamin B12 concentrations [16]. Typically, the majority of the aforementioned diseases are more prevalent among older adults than younger ones. Therefore, increased concentration of serum vitamin B12 might be a proxy for an unhealthy status in elderly adults, which could be used as a predictor of mortality.

Standard sampling procedure, representativeness of the elderly adults (especially of the oldest-old ones), and abundant information on covariables are strengths of the present study. Besides, applying the restricted cubic spline function in the model can explore their dose-response associations more precisely and avoid the arbitrary of selecting cut-off points when translating the serum concentration of vitamin B12 into the categorical variable. However, several limitations should not be ignored. First, comorbidities might affect the concentration of serum vitamin B12, such as hepatitis or chronic renal failure. However, we observed similar positive associations in the sensitivity analyses after excluding subjects with diabetes (subjects without normal glucose) and chronic renal failure (subjects without normal eGFR), which indicates comorbidities might not be a big concern in present study. Second, as the concentration of serum vitamin B12 was only measured at the baseline, its dynamic change that affected by lifestyle factors (e.g., diet and medication use) and how such change affects the mortality risk cannot be evaluated [14]. Therefore, longitudinal studies with repeated measurements are warranted in the future. Third, although folate, serum vitamin B12, and homocysteine were closely related, CLHLS was not designed to measure the other two substances, which made us unable to explore whether different concentrations of folate and homocysteine affect the interested associations. Fourth, even though results from the main analysis to the subgroup analyses were consistent, the influence of residual confounding, caused by measured or unmeasured confounders, cannot be fully ruled out. Finally, given the majority of the enrolled population in the CLHLS was the oldest-old adults, survival bias may exist, therefore generalization of the result to other age groups, areas or ethnicities needs to be done carefully.

In conclusion, our results suggested that serum vitamin B12 was associated with an increased risk of all-cause mortality among elderly adults in China. Future longitudinal studies with repeated measurements are warranted to validate our findings.

## Supplementary information

**Additional file 1**

## Data Availability

All data is stored at Peking university (http://opendata.pku.edu.cn/) and is publicly available. The datasets used during the current study are available from the corresponding author (jiangwei.sun@ki.se) on reasonable request.
