# Supplementary material for: Association between serum vitamin B12 and risk of all-cause mortality in elderly adults: a prospective cohort study
Source: BMC Geriatr. 2021 Sep 16;21:497. doi: 10.1186/s12877-021-02443-z (PMC8447618; doi:10.1186/s12877-021-02443-z)
Supplement: Supplementary file 1 — Additional file 1 [file 12877_2021_2443_MOESM1_ESM.docx]

**Association between serum vitamin B12 and risk of all-cause mortality in elderly adults: a prospective cohort study**

Kangjun Xu, Xiyu Liu, Jiaxin Liu, Yingying Zhang, Xiaohui Ding, Lin Li, Jiangwei Sun

**Stable 1.** Classification of clinical biomarkers.

| Stable 1. Classification of clinical biomarkers | | |
| --- | --- | --- |
| Biomarker | Group | Criterion |
| Total cholesterol |  |  |
|  | Desirable | <200 mg/dL |
|  | Borderline high | 200-239 mg/dL |
|  | High | ≥240 mg/dL |
| Triglyceride |  |  |
|  | Optimal | <150 mg/dL |
|  | Borderline high | 150-199 mg/dL |
|  | High | 200-499 mg/dL |
|  | Very High | ≥500 mg/dL |
| Blood pressure * |  |  |
|  | Normal | <120mm Hg and <80mm Hg |
|  | Elevated | 120-129mm Hg and <80mm Hg |
|  | Stage 1 | 130-139mm Hg or 80-89 mm Hg |
|  | Stage 2 | ≥ 140 mm Hg or ≥ 90 mm Hg |
|  | Stage 3 | ≥ 180 mm Hg or ≥ 120 mm Hg |
| Glucose |  |  |
|  | Normal | <6.1 mmol/L |
|  | Pre-diabetes | 6.1-7.0 mmol/L |
|  | Diabetes | ≥7.0 mmol/L |
| eGFR |  |  |
|  | Normal | ≥60 mL/min/1.73 m2 |
|  | Impaired | <60 mL/min/1.73 m2 |
| Abbreviations: eGFR: estimated glomerular filtration rate. | | |
| *: The first value is for systolic blood pressure, while the second one for diastolic blood pressure. | | |

**
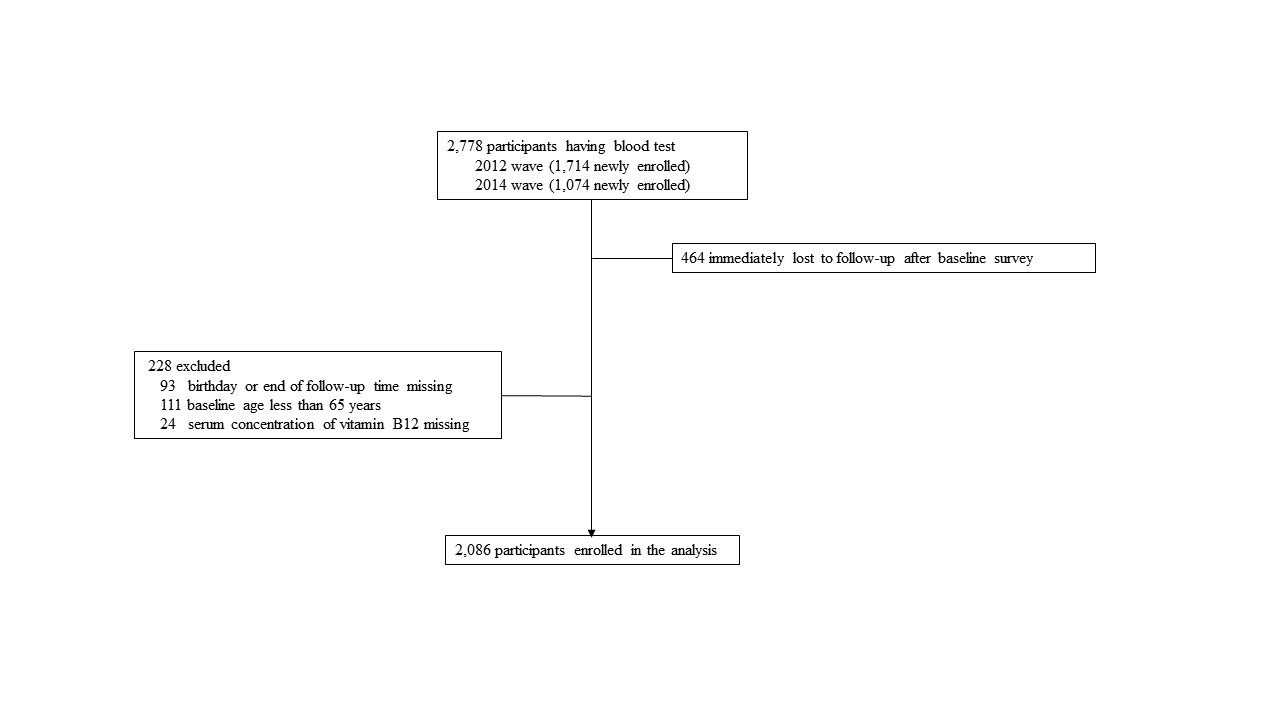
**

**Sfigure 1**. Flow chart of participant selection, a cohort study of the serum concentration of vitamin B12 with the risk of all-cause mortality among elderly adults in China, 2012-2019.


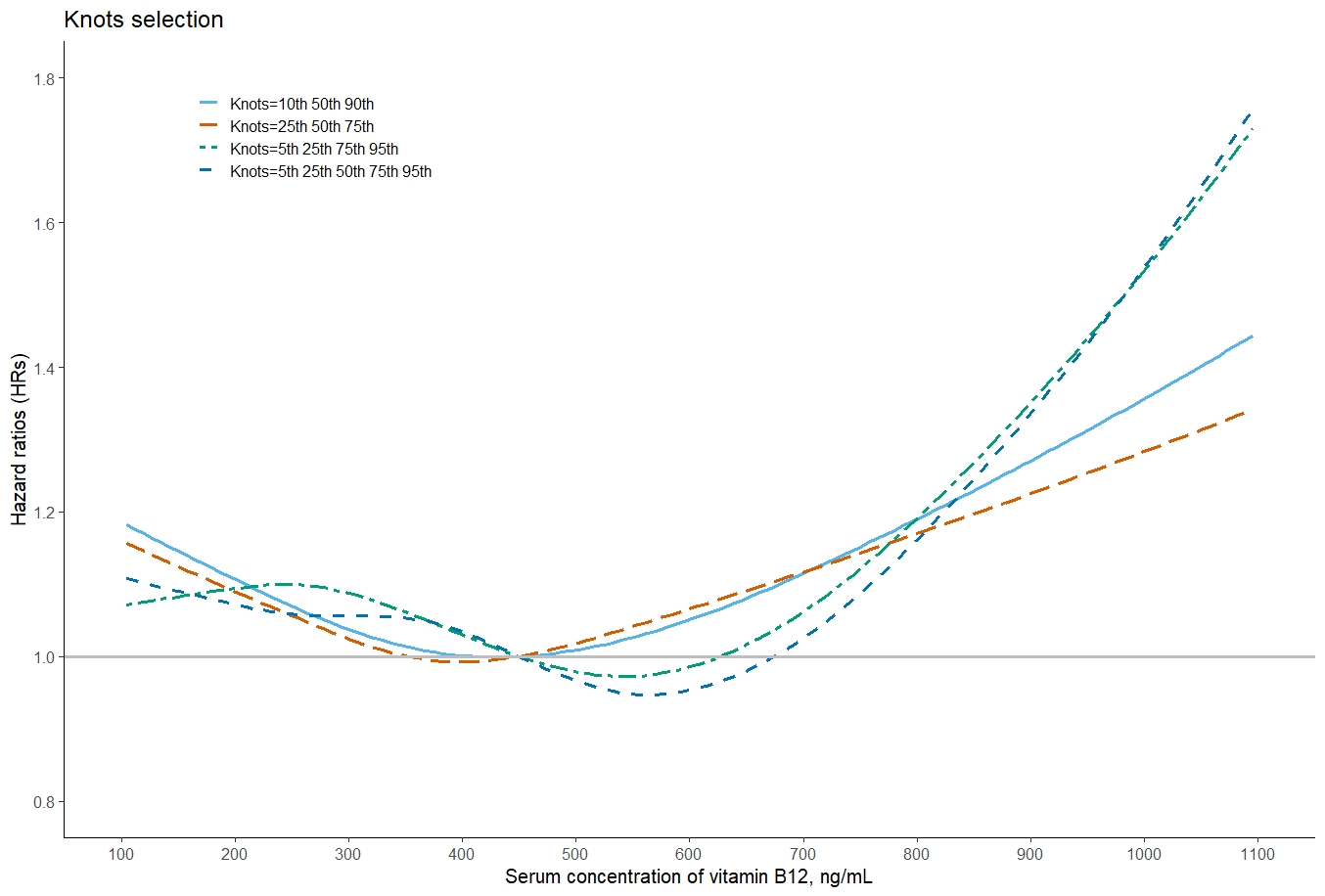


**Sfigure 2**. The adjusted dose-response associations between serum concentration of vitamin B12 and risk of all-cause mortality among the whole population, based on different knot sets in model 4. The reference value was set at 450 ng/mL.
